# Bilateral Femoral Neuropathy: A Rare Complication of Drug Overdose due to Prolonged Posturing in Lithotomy Position

**DOI:** 10.1155/2020/2352850

**Published:** 2020-03-10

**Authors:** D. Tsiptsios, D. Daud, K. Tsamakis, E. Rizos, A. Anastadiadis, A. Cassidy

**Affiliations:** ^1^Neurophysiology Department, Sunderland Royal Hospital, South Tyneside & Sunderland NHS Foundation Trust, Sunderland, UK; ^2^Neurology Department, Sunderland Royal Hospital, South Tyneside & Sunderland NHS Foundation Trust, Sunderland, UK; ^3^Second Department of Psychiatry, University General Hospital “Attikon”, School of Medicine, Athens, Greece; ^4^First Department of Urology, Aristotle University of Thessaloniki, School of Medicine, Thessaloniki, Greece

## Abstract

**Background:**

Bilateral femoral neuropathy is an uncommon complication of various surgical and nonsurgical procedures, such as pelvic/abdominal surgery or vaginal delivery. *Case Report*. We report a case of a 41-year-old male who was found unresponsive against the wall in a “lithotomy-type” position with both knees flexed at approximately 90 degrees and both hips flexed and externally rotated at approximately 90 and 60 degrees, respectively, 24–48 hours after a drug overdose (combination of dihydrocodeine, paracetamol, diazepam, and amitriptyline). During his recovery, he complained of severe bilateral proximal lower limb weakness and bilateral distal lower limb pain and allodynia. His symptoms were initially attributed to critical illness myopathy/neuropathy (CIMN). However, thorough clinical and neurophysiological evaluation revealed that his symptoms were due to severe bilateral femoral neuropathies.

**Conclusions:**

To our knowledge, this is the first reported case of bilateral femoral nerve palsy due to prolonged posturing in a “lithotomy-type” position in the context of a drug overdose.

## 1. Introduction

Bilateral femoral neuropathy is an uncommon complication of various surgical and nonsurgical procedures, such as pelvic/abdominal surgery or vaginal delivery, but rarely encountered in other scenarios or circumstances.

## 2. Case Presentation

We report the case of a 41-year-old male who was referred to our Neurophysiological Department with a suspected diagnosis of critical illness myopathy/neuropathy (CIMN) after he exhibited marked bilateral quadriceps muscle wasting with distal lower limb pain and allodynia following his recovery from coma due to a drug overdose.

Prominent fibrillations and positive sharp waves were EMGraphically recorded from the vastus medialis and vastus lateralis muscles bilaterally. Moreover, no motor unit action potentials (MUAPs) could be recruited from the aforementioned muscles. In the Intensive Care Unit (ICU) setting, such findings are usually encountered in cases of very severe critical illness myopathy.

However, tibial and peroneal motor nerve conduction studies and sural and superficial peroneal sensory nerve conduction studies were within normal limits bilaterally (Table 1). These findings were not suggestive of a large fibre peripheral neuropathy and led to a review of the clinical diagnosis of CIMN, and additional neurophysiological testing was undertaken. The findings on clinical examination were also hard to reconcile with a diagnosis of CIMN as, while the quadriceps muscles were severely wasted bilaterally, all other muscles including iliopsoas and hip adductors exhibited 5/5 motor strength. Moreover, the sensory loss was focal, with numbness and hypoesthesia over the anteromedial thighs and medial lower legs bilaterally. In addition, while the knee jerks were absent, the ankle jerks were present bilaterally.

Additional neurophysiological testing revealed that saphenous sensory nerve action potentials were absent bilaterally. Otherwise, EMG findings from iliopsoas and adductor longus muscles were unremarkable bilaterally (Table 1).

Thus, the clinical and neurophysiological findings were consistent with severe bilateral femoral neuropathies at the level of the inguinal region. It has to be stated that, while prominent abnormal spontaneous activity and unrecordable MUAPs from both quadriceps muscles are potentially suggestive of a severe underlying critical illness myopathy, it is important to realise that such EMGraphic findings can be seen in cases of severe underlying neuropathies, as in our case.

Due to the fact that a pelvic CT scan was unremarkable, with no evidence of an underlying hematoma or iliopsoas muscle swelling, and creatine phosphokinase value was within normal limits, thus excluding rhabdomyolysis and crush syndrome that could explain the patient's symptoms, his clinical history was reviewed. Interestingly, his mother had found him unresponsive 24–48 hours after a drug overdose (according to paramedics, empty packets of dihydrocodeine, paracetamol, diazepam, and amitriptyline were found around him) against the wall in a “lithotomy-type” position with both knees flexed at approximately 90 degrees and both hips flexed and externally rotated at approximately 90 and 60 degrees, respectively. This prolonged posturing seems to have resulted in severe bilateral femoral nerve palsies due to excessive nerve stretching and/or pressure under the inguinal ligament.

## 3. Discussion

Unilateral femoral neuropathy is an uncommon mononeuropathy and is usually caused by compression in the pelvis or inguinal region. Typical causes in the pelvis include compression by an iliacus or retroperitoneal hematoma, by a retractor blade during pelvic surgery, or by a pelvic mass. Typical causes in the inguinal region include compression by the inguinal ligament during the lithotomy position and by an inguinal hematoma or by other inguinal masses [1].

Bilateral simultaneous femoral neuropathy is even more uncommon. It is typically seen as a complication of either pelvic or abdominal surgery or vaginal delivery. In such instances, the proposed mechanisms of nerve injury are as follows:

- Stretching and/or prolonged compression of the nerve caused by the self-retaining retractors that can directly compress the nerve against the pelvic sidewall
- Compression of the iliac vessels causing direct ischemia of the nerve
- Prolonged compression of the femoral nerves under the inguinal ligament, or
- Excessive stretching due to excessive abduction and external rotation of the hips during lithotomy positioning [2–6]

In addition to the above iatrogenic causes, there are reports of rare cases of bilateral femoral nerve compression and ischemia due to iliopsoas hematomas [7–10] and iliopsoas swelling secondary to rhabdomyolysis [11, 12]. There are also individual reports of bilateral femoral neuropathy due to vasculitis in the context of disseminated intravascular coagulopathy [13] and due to blunt force trauma [14].

A single case of bilateral femoral neuropathy after drug overdose has also been reported [15]. On this occasion, a female had sat down on a chair with her legs stuck under a desk before then losing consciousness and falling backwards without any support for her head or arms. This prolonged hyperlordotic position resulted in a presumed stretch-induced ischemia of the nerve at the level of the iliopsoas groove.

Our case differs from the aforementioned in that our patient was found unresponsive in a different position, i.e., with both knees flexed at approximately 90 degrees and both hips flexed at approximately 90 degrees and externally rotated at approximately 60 degrees. Thus, to our knowledge, this is the first reported case of bilateral simultaneous femoral neuropathy due to prolonged posturing in a “lithotomy-type” position in the context of a drug overdose. The presumed mechanism of nerve injury is excessive nerve stretching and/or pressure under the inguinal ligament. More specifically, profound muscle relaxation due to lack of protective reflexes and muscle tone in the context of drug overdose might have resulted in excessive bilateral external hip rotation >45 degrees in a “lithotomy-type” position that also encompassed bilateral knee and hip flexion at approximately 90 degrees each. This position causes both femoral nerves to enter the thigh acutely angulated and twisted beneath the tough and inelastic inguinal ligament, leading to microvascular and/or local mechanical nerve injury. In case this position is kept for more than 4 hours, it may lead to permanent bilateral femoral nerve damage [6].

This case illustrates the importance of taking a thorough collateral history in those presenting with weakness following an admission to the ICU. A thorough neurological evaluation should always precede neurophysiological testing, as not all cases of acute or subacute lower limb weakness and sensory loss in this setting are due to CIMN. As we have described, rare neurological entities such as bilateral femoral neuropathies can also be encountered.

## Figures and Tables

**Table 1:** Neurophysiological findings.

Sensory nerve conduction studies							
Nerve and site	Onset latency	Peak latency	Amplitude	Segment	Latency difference	Distance	Conduction velocity

Peroneal R							
Ankle	2.0 ms	2.6 ms	15 *µ*V	Dorsum of foot-ankle	2.0 ms	100 mm	49 m/s
Peroneal L							
Ankle	2.1 ms	2.9 ms	12 *µ*V	Dorsum of foot-ankle	2.1 ms	95 mm	45 m/s
Sural R							
Lower leg	1.7 ms	2.6 ms	12 *µ*V	Ankle-lower leg	1.7 ms	95 mm	55 m/s
Sural L							
Lower leg	1.7 ms	2.6 ms	12 *µ*V	Ankle-lower leg	1.7 ms	95 mm	55 m/s
Saphenous R							
	No recording			Lower leg-ankle			
Saphenous L							
	No recording			Lower leg-ankle			

Motor nerve conduction studies							
Nerve and site	Latency	Amplitude	Segment	Latency difference	Distance	Conduction velocity	F-latency

Peroneal R							
Ankle	5.9 ms	2.7 mV	Extensor digitorum brevis-ankle	5.9 ms	mm	m/s	
Fibula (head)	13.0 ms	1.9 mV	Ankle-fibula (head)	7.1 ms	320 mm	45 m/s	
Tibial R							
Ankle	4.1 ms	20.0 mV	Abductor hallucis-ankle	4.1 ms	mm	m/s	53.9 msec
Tibial L							
Ankle	3.9 ms	20.7 mV	Abductor hallucis-ankle	3.9 ms	mm	m/s	53.3 msec

Electromyography							
	Spontaneous activity		MUAPs			Activation	Recruitment
	Fibrillations	PSWs	Amplitude	Duration	Phases		

Right vastus lateralis	+3	+3	No MUAPs could be recruited				
Right vastus medialis	+3	+3	No MUAPs could be recruited				
Left vastus lateralis	+3	+3	No MUAPs could be recruited				
Left vastus medialis	+3	+3	No MUAPs could be recruited				
Right iliopsoas	0	0	N	N	N	N	N
Left iliopsoas	0	0	N	N	N	N	N
Right adductor longus	0	0	N	N	N	N	N
Left adductor longus	0	0	N	N	N	N	N

PSWs, positive sharp waves; MUAPs, motor unit action potentials; N, normal.
